# An Intriguing Involvement of Mitochondria in Cystic Fibrosis

**DOI:** 10.3390/jcm8111890

**Published:** 2019-11-06

**Authors:** Maria Favia, Lidia de Bari, Antonella Bobba, Anna Atlante

**Affiliations:** 1 Istituto di Biomembrane, Bioenergetica e Biotecnologie Molecolari—CNR, Via G. Amendola 122/O, 70126 Bari, Italy; l.debari@ibiom.cnr.it (L.d.B.); a.bobba@ibiom.cnr.it (A.B.); 2 Dipartimento di Bioscienze, Biotecnologie e Biofarmaceutica, Università di Bari, Via E. Orabona 4, 70126 Bari, Italy

**Keywords:** cystic fibrosis, cystic fibrosis transmembrane conductance regulator, mitochondria, bioenergetics, oxidative stress, glucose, airway surface liquid

## Abstract

Cystic fibrosis (CF) occurs when the cystic fibrosis transmembrane conductance regulator (CFTR) protein is not synthetized and folded correctly. The CFTR protein helps to maintain the balance of salt and water on many body surfaces, such as the lung surface. When the protein is not working correctly, chloride becomes trapped in cells, then water cannot hydrate the cellular surface and the mucus covering the cells becomes thick and sticky. Furthermore, a defective CFTR appears to produce a redox imbalance in epithelial cells and extracellular fluids and to cause an abnormal generation of reactive oxygen species: as a consequence, oxidative stress has been implicated as a causative factor in the aetiology of the process. Moreover, massive evidences show that defective CFTR gives rise to extracellular GSH level decrease and elevated glucose concentrations in airway surface liquid (ASL), thus encouraging lung infection by pathogens in the CF advancement. Recent research in progress aims to rediscover a possible role of mitochondria in CF. Here the latest new and recent studies on mitochondrial bioenergetics are collected. Surprisingly, they have enabled us to ascertain that mitochondria have a leading role in opposing the high ASL glucose level as well as oxidative stress in CF.

## 1. Introduction

“The powerhouse of the cell” is surely the first memorable phrase in biology concerning mitochondria. Although this is still true, an explosion of new information about mitochondria reveals that their importance extends well beyond their time-honoured function as the “powerhouse of the cell”, essential for life.

In recent years, a large body of research has established that mitochondria are not simply static, passively producing adenosine 5′-triphosphate (ATP) for fuel, but that they sense and respond to changing cellular environments and stresses. This is the reason the research on mitochondria is feverish worldwide: an increasing number of studies place mitochondrion at the heart of cell life as well as mitochondrial dysfunction at the heart of disease, thus opening new frontiers in health and disease.

Since the 1980s, with the beginnings of molecular biology and the detection of pathogenic defects of mitochondrial DNA, it has been found that a large number of mitochondrial disorders were underlying of pathogenesis of common human diseases. This is not surprising considering that mitochondria—establishing a dynamic network intimately combined with other cellular and extracellular compartments—influence the physiology and regulate communication between cells and tissues.

Moreover, considering that mitochondrial functions respond to a number of genetic, metabolic, neuroendocrine signals, it follows that mitochondrial defects contribute to the development of various diseases by altering complex cellular and physiological functions. In fact, it is now ascertained that many seemingly disconnected diseases have tangled roots in dysfunctional mitochondria. At the same time, it is also true that modern research has also endowed us with the knowledge on how to optimize their function, which is of critical importance to our health and longevity.

Reflecting on the fact that mitochondria have received increasing attention, especially in recent decades, we argue that their increasing relevance to modern medicine [1,2], is attributable to the convergence of key signalling pathways and biological processes onto the mitochondrion. Moreover, since mitochondria have an enormous potential to influence health, the optimizing of their metabolism could really to be the focus of an effective therapeutic treatment. Consequently, it is no wonder that mitochondria failure risks the collapse of most crucial cellular functions: this is the reason for which mitochondrial dysfunction is strictly connected to the aging phenomenon and numerous human diseases. It happens that biomedical scientists frequently ‘fortuitously’ encounter mitochondria during the natural development of their research program, as well as recent studies have brought to light unsuspected pathophysiological mechanisms involving this organelle. However, it is not yet clear whether mitochondrial dysfunction is a trigger for or a consequence of disease. For example, no one would have suspected, mitochondrial involvement in cystic fibrosis (CF) progression, an inherited disease characterized by alterations in the cystic fibrosis transmembrane conductance regulator (CFTR) protein, which plays a role in regulating hydrosaline balance on many surfaces in the body.

At the beginning of the research about the direct involvement of mitochondria in CF, a consideration is worthy of note: it is a question of building a cathedral in a desert. At the moment, the small steps that research is making in this direction are almost invisible and surely researchers, who are now laying the foundations to promote it, will not reap the benefits. However, every step will have great importance for who will outline the molecular mechanisms responsible for the implication of these cellular organelles in CF in the future. Discovering the invisible traces of the thousands of metabolic reactions in which mitochondrial enzymes are involved, can reveal the secrets of the CF cell and find the points of attack on which to act to obtain therapeutic responses.

That’s why the authors of this Review embarked on this venture: to collect all the data—at the moment very few—available on the involvement of mitochondria in CF to understand how to proceed with exploration of mechanism underlying regulation of mitochondrial function with the last hope of glimpsing viable paths for future therapies.

## 2. Cystic Fibrosis: News on the Disease

CF is the most common and severe multisystem genetic disease among Caucasians and is estimated to affect about 36000 individuals in the European Union [3], and approximately 80,000 people in the world [4], with an incidence of 1 in 2500 Caucasians [5,6]. 

CF is caused by a defective gene *cftr* [7] that encodes for a protein called CFTR. The CFTR is a cAMP-regulated anion (Cl^−^) channel. Playing crucial roles in both absorption and secretion [8,9], it is found primarily in wet epithelia, consistent with the symptoms that define CF. Besides being directly involved in the transport of chloride, it participates in the transport of other ions, as sodium and bicarbonate, so controlling salt and water transport across epithelial cell membranes of many tissues [10]. Indeed, CFTR protein is expressed in various epithelial cells lining many organs including the lung, pancreas, liver, the digestive and reproductive tracts [8,9,11,12]. However, CFTR protein is also present in non-epithelial cells from blood, brain, heart, liver, kidney and other tissues [13,14,15,16,17,18,19]. Additionally, CFTR is transcribed in the central, peripheral, and enteric nervous systems [20]. Although many organs are affected in CF, the most severe pathological consequences are lung-associated. For this reason the paper will be focused on the cell affected by CF in airways. 

### 2.1. CFTR Protein

#### 2.1.1. Domain Structure of CFTR 

The CFTR protein belongs to the superfamily of ABC (ATP Binding Cassette) transporters proteins that have a characteristic modular structure consisting of two hydrophobic transmembrane domain, usually made of six membrane-spanning α-helices, and two cytosolic nucleotide-binding domains (NBD1 and NBD2) [21]. In addition, CFTR is unique among ABC transporters to have a regulatory (R) domain that links two homologous halves [22]. CFTR activation requires both ATP binding to the interface between NBD1 and NBD2 and protein kinase A (PKA)-mediated phosphorylation of the R domain [23,24,25,26]. 

CFTR activity (i.e., channel opening and closing) is controlled both by phosphorylation and dephosphorylation processes, via protein kinases and phosphatases, and by cellular ATP levels. It is also known that CFTR functional expression is regulated by interactions of CFTR protein with several proteins [27,28,29,30,31,32] in order to form a macromolecular complex.

#### 2.1.2. CFTR Synthesis and Trafficking 

In order to ensure that its functioning on the apical membrane is optimal, CFTR protein must be synthesized, folded, and transported in a correct manner. For this to happen, it must be subjected to a stringent quality control which removes any misfolded protein that could fail to function properly [33]. The normal biogenesis of CFTR starts with the translation of the CFTR protein in the rough endoplasmic reticulum (ER). Simultaneously a glycan (sugar molecule) is attached to a nitrogen atom of the protein in a process called N-linked glycosylation. Several proteins, as the chaperones, are involved in the correct folding of CFTR [4,34,35].

An important cytosolic chaperone, calnexin [36], interacting with the immature form of CFTR in ER, favours its proper folding. After folding in the ER, the CFTR is submitted to the ER-associated degradation process, involving the ubiquitin proteasome system [33]. Now, the aberrantly folded CFTR proteins undergo polyubiquitylation, removed from the ER membrane and degraded by a proteasome in the cytoplasm [37], whereas the correctly folded CFTR protein is sent to the Golgi by the coat protein complex II (COPII). Thanks to COPII, CFTR protein maintains the right structure, conformation, and protein-protein interactions. Next, within the Golgi, CFTR assumes its mature form and it is moved to the apical membrane via clathrin-coated vesicles [38]. In the plasma membrane, CFTR has a half-life of about 12–24 h; then it is internalized by clathrin-coated endosomes and either sent back to the plasma membrane or degraded within lysosomes [33].

#### 2.1.3. CFTR Function in Physiological Conditions

In the lung, CFTR protein is expressed on the apical membrane of the cells lining the airways where it functions as a regulated chloride ion channel to maintain the balance of salt and water on lung surface. In fact, through an ATP-ase activity, requiring the use of an ATP molecule, CFTR protein favours the passage of chloride (but also of other electrolytes, such as sodium) from the inside to the outside of cells, with consequent secretion of water [39]. 

**Figure 1:**
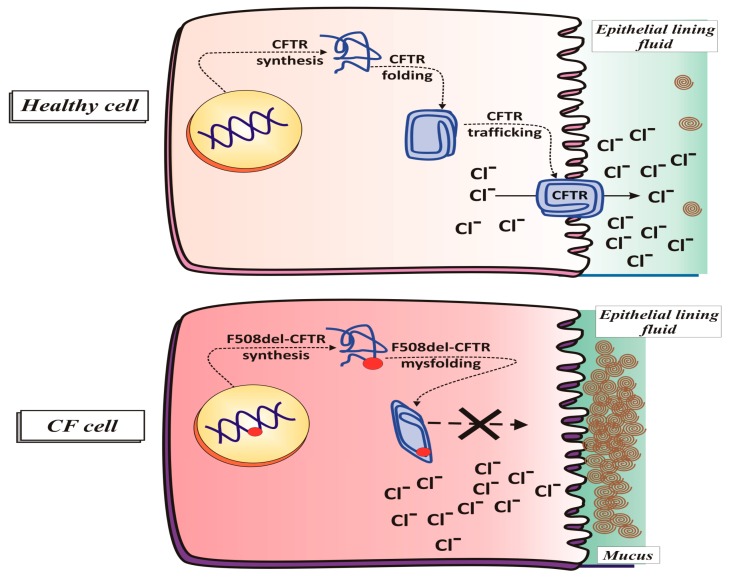
CFTR activity in the cell. In a ***HEALTHY CELL***, a correct insertion of normal CFTR on the membrane allows for the ion movement across airway epithelium; in a ***CF CELL***, transport Cl^−^ ions does not occur due to the mutated-CFTR channel protein (F508del-) inability for reaching the plasma membrane.

**Figure 2:**
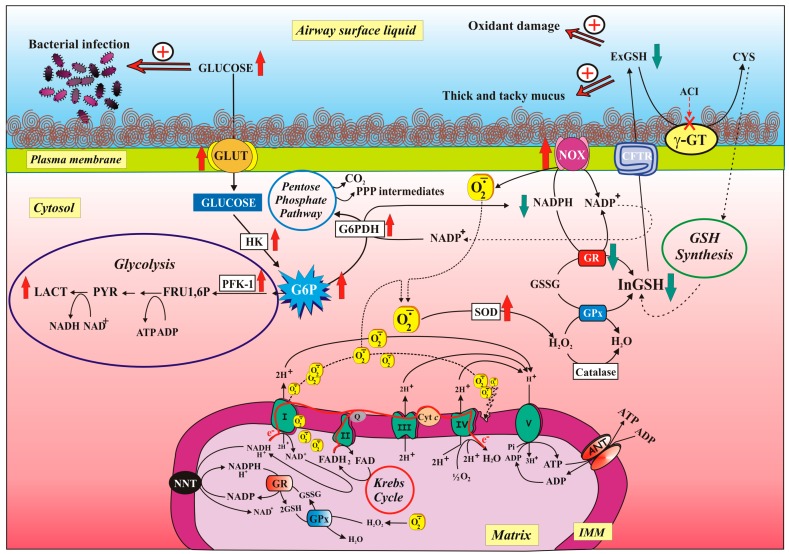
An overview of both the GSH turnover and the principal glucose related metabolic pathways: modulation of ROS and glucose levels in airway surface liquid and fight oxidative stress in cystic fibrosis cells. *Main abbreviations:* ACI, Acivicin; ANT, adenine nucleotide translocator; CYS; Cysteine; CI, Complex I or NADH-ubiquinone oxidoreductase; II, Complex II or succinate-ubiquinone oxidoreductase; III, Complex III or ubiquinone–cytochrome-c oxidoreductase; IV, Complex IV or cytochrome-c oxidase; V, Complex V or FoF1 ATP synthase; GLUT, Glucose Transporter/s; G6P, Glucose-6-phosphate; G6PDH, glucose-6-phosphate dehydrogenase; exGSH, extracellular GSH; inGSH, intracellular GSH; GPx, glutathione peroxidase; GR, glutathione reductase; GSSG, oxidized glutathione; γGT, γ-glutamyltransferase; HK, Hexokinase; H_2_O_2_, hydrogen peroxide; IMS, intermembrane mitochondrial space; MIM, mitochondrial inner membrane; NOX, NAD(P)H oxidases; O_2_^−^^•^, Superoxide anion; OMM, outer mitochondrial membrane; PPP, pentose phosphate pathway; ROS, reactive oxygen species; SOD, Superoxide dismutase.

In addition, CFTR has been implicated in the secretion of bicarbonate, necessary for the bactericidal activity of the fluid that wets the airways, and also in the glutathione (GSH) efflux from cells [39], then implicating a role for CFTR in the control of oxidative stress in the airways [40]. Moreover, of note is the fact that CFTR protein regulates the activity of other chloride and sodium channels at the cell surface epithelium [39]. The balance between these transport functions is thought to lead to an optimal airway surface liquid (ASL) volume to promote ciliary clearance of mucus and bacteria (see Figure 1 and Figure 2). 

#### 2.1.4. CFTR Function in Pathological Conditions (Cystic Fibrosis)

In CF, the gene mutation determines the production of a defective CFTR protein or even prevents its synthesis, with disruption of ion transport and the consequence that the secretions are poor in water, therefore dense and not very flowing, thus preventing effective ciliary activity [41]. In fact, mutated CFTR is not able to maintain the proper chloride levels, leading to an increase in intracellular chloride levels and a decrease in extracellular ones (see Figure 1). In particular, in CF the impairment of chloride transport is coupled with an increased sodium absorption by the airway epithelial cells, followed by an excessive movement of water into the airway epithelial cells. This represents a key step in the pathogenesis of CF lung disease, i.e., airway surface liquid dehydration, leading to thick and sticky mucus formation [39], ineffective mucociliary clearance [42] and exaggerated inflammation in response to infection. Chronic inflammation of the lung, as a consequence of persistent bacterial infections by several opportunistic pathogens, represents the main cause of morbidity and mortality in patients affected by CF [43]. Moreover an increase in anionic polyelectrolytes, including DNA derived from bacteria and lysed inflammatory cells, contributes to thicken the mucus [42].

In addition to the epithelial cell defects, a growing body of evidence has emerged indicating that neutrophils and macrophages, being central to the infectious and pulmonary pathology, account for the majority of CF mortality [44]. The intrinsic effect of CFTR deficiency in neutrophils and macrophages appears to be an inability to effectively kill bacteria [45]. Recent works prove that autophagy, a process clearing pathogens and dysfunctional protein aggregates within macrophages, is impaired in CF patients and CF mice, as their macrophages exhibit limited autophagy activity. This is the reason for which the study of microRNAs (Mirs), and other noncoding RNAs offers new therapeutic targets, with the aim to elucidate the role of Mirs in dysregulated autophagy-related genes in CF macrophages, and then target them to restore this host-defense function and improve CFTR channel function [46]. Furthermore, CF neutrophils are deficient in chlorination of bacterial components due to a limited chloride supply to the phagolysosomal compartment. About this, CFTR channel expression in neutrophils and its dysfunction affect neutrophil chlorination of phagocytosed bacteria [47]. 

Of note, these particular aspects of the disease, besides to have a clinical value because a cause-and-effect relationship has been established between ion transport and gene expression in CF immune cells, also transform the way we look at CF, which is now classified as an immune deficiency disorder [44,48].

#### 2.1.5. Classes of Mutations

To date, more than 2000 mutations of the *cftr* gene have been described (Cystic Fibrosis Mutation Database; www.genet.sickkids.on.ca), although the most frequent mutation causing CF disease is the deletion of three base pairs in both copies of the gene that causes the loss of phenylalanine residue at position 508 of the CFTR protein (F508del) [49]. Different mutations prompt varying effects on CFTR function, resulting in diverse phenotypes of the disease. 

Mutations known to affect the CFTR protein have been divided into classes (I to VI). The mutations belonging to classes I, II and III most alter the fate of the protein, not allowing any production (class I) or producing a very defective protein (class II and III); those of class IV allow the synthesis of a defective protein but capable of carrying out its function, even if in reduced measure; those of class V lead to a reduced amount of functional CFTR protein. Lastly, class VI mutations reduce CFTR stability, causing to accelerated channel removal from the plasma membrane [37].

##### F508del CFTR

Nearly 70–90% of the CF patients carry the allele F508, where phenylalanine at position 508 (F508del CFTR) in the NBD1 domain of the protein is lost leading to misfolding of the protein causing the most severe defect, that is the mistrafficking of CFTR protein that remains trapped in the endoplasmic reticulum and is subsequently degraded, before reaching the membrane.

However, in some CF airway cells a negligible expression of F508del CFTR can be detected at the cell surface due to the fact that ER retention is not complete [50,51]. Furthermore, the F508del-mutation reduces its apical membrane half-life [52] by accelerating its endocytic retrieval from the plasma membrane and its consequent degradation [53].

### 2.2. Clinical Trials in Cystic Fibrosis 

#### 2.2.1. Treatments of CF Symptoms

Quality of life as well as survival are affected by this disease. Although life expectancy has improved [54,55], current treatments for CF are neither preventive nor curative. In fact, since its recognition CF has been treated symptomatically.

One of the most common treatments is the inhalation of osmotic agents which, assuring an increase in mucociliary clearance [56], leads to better lung function within CF patients [57].

Another common treatment for CF patients is the inhalation of an enzyme deoxyribonuclease I that cleaves DNA in order to reduce the viscosity of the mucus [56,58]. Another treatment helping to slow the progression of CF disease is the chest physiotherapy [59]. 

Furthermore, since there are a wide variety of bacterial infections that can take hold in CF patients’ lungs, there is also a large amount of antibiotic treatments focus on the controlling pulmonary infections. Lastly, lung transplantation is a complex, high-risk, potentially life-saving therapy for the end-stage of CF [60].

#### 2.2.2. Latest Breakthrough Therapies

Daily, expensive drug-based options for treating the downstream effects of the CFTR gene defect, having the potential to improve survival and quality of life in patients with CF, are the major areas of focus of clinical trials.

In fact, the findings that restoration of small amounts of functional CFTR protein (20–30% of normal levels) [61] can greatly ameliorate the disease severity, have prompted researchers to identify modulator molecules able to rescue the CFTR defect thus restoring its folding, trafficking, and insertion into the plasma membrane (correctors) and/or improving its regulated function once its insertion on the surface (potentiators). Correctors are small molecules designed to increase the availability of CFTR protein at the apical membrane of epithelial cells and stay there longer (see Figure 1). Two F508del-CFTR corrector molecules, VX-809 and 4,6,4′-trimethylangelicin (TMA), were used in our studies in order to improve mitochondrial impairments, associated with the ΔF508 mutation (see below, Section 5.2.1) [61,62].

However, the combined use of corrector and potentiator may work on residual function allowing more chloride to flow through cell epithelium and reduce the symptoms of CF. 

To these molecules acting as modulators, others recently are added: the amplifiers. They are CFTR modulators that improve translation of CFTR mRNA to increase CFTR protein production. However, amplifiers are not yet available.

Other alternative approaches have been proposed, such as inhibiting ENaC activity [63] or activating an alternative chloride channel [64].

Lastly, since CF is a recessive genetic disorder, the addition of a single copy of the properly functioning CFTR gene into affected CF airway cells could represent the only rational and feasible way to prevent or treat CF airway disease for all CFTR mutation classes [65]. 

## 3. Lung: Information and Facts

The primary function of the respiratory system is to exchange oxygen and carbon dioxide. The inhaled oxygen enters the lungs and reaches alveoli and the bloodstream, by which it is transferred to all the tissues and organs of the body. Besides the skin, the lung is the only organ that is in direct contact with the external environment. As a consequence, it is constantly exposed to inhaled microbes, allergens and particulate material, which must be cleared without inducing inflammation, so as to maintain homeostasis. Airway epithelium, functioning as a physical barrier against external environmental insults, protects the lungs. Therefore, structurally and/or functionally damage to the epithelium may contribute to inflammation establishment and to alteration of repairing process following an injury. 

### 3.1. Lung: A Metabolically Active Organ 

Although the lung is often not considered a metabolically active organ, biochemical studies demonstrated that glucose utilization in lung [66] surpasses that of many other organs, including the heart, kidney, and brain. As for most tissues, glucose represents the primary source of energy also for the lung. In fact, glucose oxidation has been estimated to be 40–50 μmol/(h·g) of dry lung weight, which is a value equal to or greater than most other metabolically active organs [67,68]. 

The first step in glucose metabolism consists in its phosphorylation by hexokinase (HK) that occurs inside the cell. This process has the double advantage of trapping glucose inside the cell and providing a transmembrane concentration gradient to uptake more glucose (see below, Section 3.4).

In the cell, glucose metabolism involves four pathways: Krebs cycle and oxidative phosphorylation (OXPHOS) occurring in the mitochondria, and glycolysis and pentose phosphate pathway (PPP), which take place in the cytoplasm. Glycolysis and the Krebs cycle provide reducing equivalents for OXPHOS and finally produce ATP and NADH, while PPP, of which glucose-6-phosphate dehydrogenase (G6PDH) is the first and rate-limiting enzyme [66] and NADPH is the main product, mainly plays an important role in the fight against oxidative stress. Therefore, G6P is at the nexus of the PPP oxidative arm, glycogen synthesis—via conversion to glucose-1-phosphate—and glycolysis. The predominant fate of G6P depends on the cell type and metabolic demand. 

In the lung, mitochondria preferably use substrates derived from glucose, such as pyruvate, for the production of oxidative energy, however, other energy sources are also used, including fatty acids, intermediates of the Krebs cycle, glycerol-3-phosphate and glutamate [69]. With classical teaching, pyruvate is metabolized in mitochondria under aerobic conditions. However, it is interesting to note that pyruvate conversion—by one additional cytosolic enzymatic reaction—to lactate in the cytoplasm appears to be largely independent of oxygen concentration, as levels have been shown to increase only marginally when alveolar PO_2_ levels are significantly reduced [61]. This suggests that the lung may have evolved to use aerobic glycolysis as a means of minimizing local oxygen consumption, thus improving overall supply of oxygen delivery to other tissues.

Furthermore, it has been proposed that lactate production could serve as an energy source for lung cells, particularly, for those that have not adequate access to nutrients in the pulmonary circulation. This is not surprising if we consider the existence of monocarboxylate transporters and lactate dehydrogenase LDH isoforms in mitochondria in different healthy tissues as previously reported [70] and supported by the MitoCarta list [71].

If true, then the healthy lung would have used a strategy often attributed to cancer cells, in which lactate secretion by the primary tumour cell is important to support the activities of other cells (e.g., stromal cells) in the tumour microenvironment [72]. Moreover, lung mitochondria also have a unique and advantageous metabolic adaptation to aerobic OXPHOS, since the lung possesses its own isoform of the complex IV of the electron transport chain (ETC), cytochrome c oxidase (COX subunit IV-2), present in all lung cells, more oxygen sensitive, thus making the pulmonary COX more two-fold active (oxygen-binding) than COX in other tissues [73].

### 3.2. Lung Redox Homeostasis 

As commonly indicated, molecular oxygen is a prerequisite for the life of all aerobic organisms and is essential for its many roles in human physiology. However, it is known that high concentrations of oxygen or its metabolites, i.e., reactive oxygen species (ROS), are able to cause cellular lesions and contribute to the pathogenesis of the disease. In particular, the lung is exposed to several thousand litres of air per day that carry a very large number of compounds with oxidative potential, including air pollution, pollen, and particulate matter. Although bigger particles are efficiently cleared by the nose and upper airways, fine particles can easily access the lower airways and promote greater airway oxidation and inflammation [74,75]. In order to effectively regulate the biological actions of exogenous and endogenous ROS, various enzymatic and non-enzymatic antioxidant defence systems are present in all types of lung cells to provide adequate protection against their harmful effects. 

An increase in ROS production or a reduction in the ability to eliminate ROS can destroy redox homeostasis, leading to an overall increase in intracellular ROS levels or oxidative stress. Prolonged activity of cells at abnormal levels of ROS causes genetic mutations, which make them well adapted to oxidative stress. Thus, the cells that survive intrinsic oxidative stress mobilize a series of adaptive mechanisms, which activate ROS-scavenging systems to combat oxidative stress [76].

In this regard, it is important to reflect on what is meant by oxidative stress. Oxidative stress is considered as an imbalance between pro- and antioxidant species, which results in molecular and cellular damage. This definition lends itself to the idea that in reality there is a particular balance and that deviations from it can affect homeostasis and potentially cause or worsen the disease. Therefore, many studies, albeit with disappointing results, have been focused on restoring this “balance” through the use of antioxidants. A problem that should not be underestimated is the difficulty the researcher encounters in distinguishing when, in a given pathological process, oxidative stress is a guiding factor or simply an epiphenomenon. 

Acute and chronic lung diseases are thought to be associated with an increase in oxidative stress, evidenced by greater irreversible oxidative changes in proteins or DNA, mitochondrial dysfunction and altered expression or activity of NOX (NAD(P)H oxidases) enzymes and antioxidant enzyme systems. However, it is right to consider that, besides these presumed damaging effects of NOX-derived ROS, NOX-family enzymes participate in other cell functions such as cell proliferation, differentiation, etc. [77,78].

The most accepted hypothesis is that in CF the excessive production of ROS (probably by neutrophils activated during infection cycles) overloads the antioxidant defences and oxidises the components of the lung cell membrane, thus contributing to lung dysfunction, following repeated episodes of infection. In particular, it has been observed that patients with severely impaired pulmonary function had significantly elevated plasma concentrations of lipid hydroperoxides [79,80], suggesting that lipid peroxidation is closely associated with the decrease in pulmonary function seen in CF. In addition, markers of oxidative stress were present in many CF patients, even though they had normal concentrations of circulating antioxidants, thus suggesting that normal levels of antioxidant defences are insufficient to protect against the oxidative stress. As a result, cumulative oxidative lung damage contributes to the progressive decrease in pulmonary function observed in these patients [81].

### 3.3. Airways Surface Liquid: Characteristics and Functions 

The common feature of chronic airway diseases in humans is mucociliary dysfunction. 

Briefly, mucociliary clearance is an important primary innate defence mechanism that protects the lungs from deleterious effects of inhaled pollutants, allergens, and pathogens. The mucociliary apparatus consists of three functional compartments, namely, the cilia, a protective layer of mucus, and an ASL layer, which work in concert to remove inhaled particles from the lung. 

In this context, we will only report information about ASL that is pertinent to what will be discussed later in the paper. 

The ASL, initially recognized for its property of reducing the surface tension facilitating alveolar compliance, is now appreciated as a first line of defence against inhaled chemical agents and pathogens [82]. The importance of ASL for the healthy function of the epithelium mainly concerns the correct function of the cilia, which would be unable to beat if ASL was absent, as well as mucociliary transport would be absent. As a consequence, various defensive mechanisms in the airway mucosa would be defective. Therefore, everything related to the ASL, i.e., the volume, pH, ionic and nutrient content is important in regulating antimicrobial activity, ciliary function and mucociliary transport of the airway. In particular, the water content of ASL is controlled by the regulation of ionic transport mediated by chloride channels (CFTR and a calcium-activated [alternative] chloride channel) and the epithelial sodium channel ENaC [43,83]. In detail, airway epithelia absorb Na^+^ through ENaC and secrete Cl^−^ through the CFTR anion channel. This balance maintains adequate hydration of superficial airway fluid to permit an effective elimination of the mucus, required to conserve sterility of the lung.

As evidence of the functional importance of these channels, we can consider that a series of human pathologies, including CF [84], chronic bronchitis [85], chronic obstructive pulmonary disease (COPD) [86], and pulmonary edema [87], are associated with the impairment of epithelial ionic transports. 

Indeed, in normal airways, CFTR and the ENaC are perfectly functioning [88]. The combination of Cl^−^ secretion and reduced Na^+^ reabsorption favours a healthy ASL ion composition and depth, which enables effective ciliary function for appropriate mucociliary clearance [88]. 

In chronic airway diseases, such as CF, i.e., when CFTR is absent or dysfunctional and ENaC is no longer regulated, hyperabsorption of Na^+^ and an increased driving force for fluid reabsorption [88] occurs. Furthermore, the ASL depth is reduced, the mucosal glands are hypertrophic and excess mucus is secreted [88]. The excessive production of viscous mucus impairs mucociliary clearance, resulting in airflow obstruction and bacterial colonization of the lungs [88].

### 3.4. Glucose Movement Across the Airway Epithelium

Another task of the ASL is to maintain differential glucose concentration. Glucose is exclusively supplied to the airways by circulating blood, and reaches the basolateral side of epithelial cells, where it can be absorbed. Levels of glucose in the lungs are tightly regulated and are up to 12 times lower in the lung ASL than in circulating levels (differential glucose concentrations between the ASL [~0.4 mM] and the blood/interstitium [5–6 mM]) [89,90].

The glucose concentration in ASL is kept low—an important condition to protect the lung from infections—both by the action of facilitative glucose transporter/s (GLUT/s) and by its subsequent metabolism occurring in lung epithelial cells [91]. 

Consistently, Bearham et al. [90] hypothesised that movement of glucose in the airway largely depends on its intracellular concentration, which is regulated by the activity of hexokinase. Low intracellular glucose preserves a driving force for glucose to enter the cell. However, if intracellular glucose concentrations rise to the same or higher values of glucose present in ASL, this would favor luminal efflux of glucose. 

Since an increase of glucose in the ASL has been associated with an increase in respiratory tract infections in airway disease [92], the knowledge of the dynamics underlying glucose movement across the airway epithelium is fundamental. Recently Bearham et al. [90] showed that inhibition of apical GLUT uptake with Cytochalasin B increased apical glucose accumulation, indicating that without the contribution of GLUT-mediated absorption, glucose levels in ASL are likely to increase further in response to proinflammatory mediators. To confirm this, the clinical observations show that, in humans, airway inflammation is associated with increased ASL glucose concentrations [92]. Therefore, maintaining a low level of ASL glucose is essential for preserving airway sterility.

To confirm that the low concentration of glucose in ASL is a key element in lung defence against infection, a study conducted on patients in intensive care showed that patients with high ASL glucose concentrations were more likely to acquire respiratory infections—particularly with methicillin-resistant *Staphylococcus aureus*, which uses glucose as a growth substrate—compared to those with normal ASL glucose concentrations [93]. Consistently, diabetic patients with and without chronic lung disease are at increased risk of respiratory infection.

However, to date, it has not yet established how the human airway epithelium is able to regulate the concentration of glucose in ASL. Since in healthy subjects, in which blood glucose increase was induced experimentally, glucose concentration in ASL increased [94,95], it appears that glucose moves through the epithelium along the concentration gradient by paracellular diffusion. When the experimental hyperglycemia is reversed, the ASL glucose concentration decreased, suggesting that glucose is removed from the ASL by absorption by cells against a transepithelial glucose concentration gradient since the ASL glucose always remained lower than the concentrations of blood glucose [94,95]. Consistently, it has been observed by [96] that CF-related diabetes is associated with a more rapid decline in lung function. Many evidences indicate that diabetes and hyperglycemia, even in non-CF patients, are associated with reduced lung function compared to control non-diabetic subjects [97]. In addition, high blood glucose concentrations, particularly in CF patients, have been associated with elevated airway glucose levels and an increased risk of bacterial infections [for review see [96]]. Moreover, hyperglycemia may disrupt the benefits of CFTR correctors on airway repair.

## 4. Mitochondria

### 4.1. Mitochondria: A Short Brief and Essential Presentation

Although mitochondria are far more than just power suppliers—having them main roles in apoptosis, calcium homeostasis and oxygen sensing [98,99,100]—they remain famous for producing ATP via OXPHOS. 

**Figure 3:**
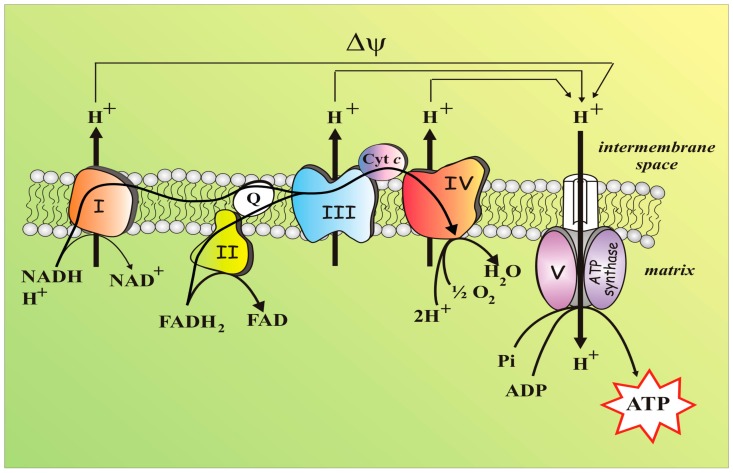
A schematic representation of the mitochondrial respiratory chain. Main abbreviations: ANT, adenine nucleotide translocator; I, Complex I or NADH-ubiquinone oxidoreductase; II, Complex II or succinate-ubiquinone oxidoreductase; III, Complex III or ubiquinone–cytochrome-c oxidoreductase; IV, Complex IV or cytochrome-c oxidase; V, Complex V or FoF1 ATP synthase; ∆Ψ, Mitochondrial membrane potential.

The mitochondrion, a semi-autonomous organelle with an own maternally inherited genome as well as the full apparatus for transcription/translational processes [101], is enclosed within outer and inner membranes that identify the two compartments of intermembrane space (IMS) and matrix. The inner membrane (IMM), which protrudes into the matrix with the cristae, harbours the OXPHOS enzyme complexes, which altogether form ETC or respiratory chain (Figure 3). In the matrix, the enzymatic reactions of the tricarboxylic acid cycle (TCA) produce NADH and FADH_2_ which act as electron-carriers to the respiratory chain complexes, thus inducing oxygen consumption. As a result, the oxidation of food-derived, high-energy molecules, which starts into the cytoplasm and culminates with electron flow along the ETC and oxygen consumption, allowed the chemical energy being trapped into a trans-membrane electrochemical potential (ΔΨ) [102]. Any defect in the energy flow will alter mitochondrial homeostasis and induce pathological conditions.

The ETC consists of four protein machines (I–IV), which through sequential redox reactions undergo conformational changes to pump protons from the matrix into the IMS. In details, nutrients (e.g., glucose, fatty acids and aminoacids) are degraded to small metabolites (e.g., pyruvate, acetyl-CoA, oxaloacetate, 2-oxoglutarate) which are oxidized by the enzymes of the TCA cycle where electrons, made available in the decarboxylation reactions, are transferred to NAD^+^ producing NADH.

Complex I (mtCx-I), i.e., NADH:ubiquinone oxidoreductase, also known as Nicotinamide adenine dinucleotide (NADH) dehydrogenase—a sophisticated microscale pump consisting of 45 core subunits, whose biogenesis requires an army of assembly factors [103,104]—thereafter oxidizes NADH and induces the release of electrons that flow through ubiquinone Q to generate ubiquinol. The ubiquinone Q can further receive electrons from other sources, i.e., Complex II (succinate dehydrogenase), electron transfer flavoprotein oxidoreductase, dihydroorotate dehydrogenase, and FAD-linked glycerol-3-phosphate dehydrogenase [105]. Electrons then proceed through cytochrome *c* and Complex III up to Complex IV where the terminal electron acceptor, i.e., O_2_, is reduced to H_2_O (see Figure 3). This flow of electrons along the respiratory complexes is an energetically favourable process sustained by the difference in the redox potentials of NADH (Eo′ = −340 mV) and O_2_ (Eo′= +810 mV). According to Peter Mitchell’s chemiosmotic theory, the electron flow is coupled to the pumping of protons through Complexes I, III, and IV into the intermembrane space and the released energy is temporary stored in the so-called protonmotive force. This energy reservoir allows ATP to be synthesized from ADP and free phosphate when protons move down this gradient at the level of the F_1_F_O_-ATP synthase. The newly synthesized ATP can be translocated into the cytosol through the adenine nucleotide translocase (ANT) (Figure 3). 

The ΔΨ, generated at the IMM level, not only provide cell with newly synthetized ATP, but it is a crucial feature of healthy mitochondria [106], being the driving force for other mitochondrial processes, such as mitochondrial protein imports [107] or the key factor that underlies any changes of mitochondrial behaviours in response to mitochondrial dysfunction. In addition, the mitochondrial matrix is central to metabolism, as OXPHOS, the citric acid cycle, fatty acid oxidation, the urea cycle and the biosynthesis of iron sulphur centres and can take place there. 

A consequence of mitochondrial respiration is the generation of unpaired electrons. Molecular oxygen can be reduced to H_2_O by only one electron at a time, but it may happen that spurious electrons, mainly originating from complex I and III, reduce O_2_ to produce superoxide anion (O_2_^−•^) [108]. O_2_^−•^ is an highly ROS that is quickly dismutated to H_2_O_2_, a signalling molecules still belonging to ROS, which is endowed with a longer half-life and increased capacity to cross biological membranes. 

In addition to being the main source of ROS, mitochondria also contain the cell’s antioxidant defences [109]—such as superoxide dismutase, peroxidases and catalase, and small molecules such as GSH—to curb the damaging effects of ROS, thus protecting the cell. This makes mitochondrion a central player in cellular redox homeostasis. It follows that subtle changes in respiratory chain capacity, substrate supply, GSH levels and membrane potential could determine conditions predisposing towards diseases as well as in genetic disorders.

### 4.2. Mitochondria, An Essential Part of the Redox Balance 

It is undisputed that mitochondria, playing a central role in the regulation of cellular bioenergetics, respond to changes in the environment caused by hormones, nutrients, partial oxygen pressure, oxygen amendments and others [110] and are essential for cell viability. It follows that the mitochondrial redox control affects the redox balance of the entire cell [111] that in turn affects all cell metabolism, as we will see below. The cytosolic redox state strictly depends on the reduced/oxidized ratio of specific cofactors—NADH/NAD^+^, NADPH/NADP^+^ and glutathione (GSH)/glutathione disulfide (GSSG)—which are involved in maintaining cell homeostasis and counteracting oxidative stress. The ratio of these redox/active cofactors is greatly influenced by the energy status of the cell, i.e., availability of energy substrates and ATP, as well as by any alteration in physiological conditions. 

About the NADH/NAD^+^ pair, under basal condition, the oxidized form, i.e., NAD^+^, prevails over the reduced one [112] and as such it is used in the glycolysis within the glyceraldehyde-3-phosphate dehydrogenase reaction which leads to the production of 1,3-diphospho-D-glycerate and NAD(P)H. Thus, the reduced state of this cofactor is closely linked to the pathways that contribute to the synthesis of ATP, i.e., glycolysis and OXPHOS. Indeed, full oxidation of glucose to CO_2_, occurring first during glycolysis and then in the citric acid cycle, has the effect of reduce the electron acceptor NAD^+^ to NADH. Due to the continued demand of NAD^+^, it is regenerated by oxidation of NADH. Cytosolic NADH can be directly oxidized to NAD^+^ in the last glycolytic reaction which converts pyruvate to lactate, otherwise its electrons can cross the mitochondrial membrane via enzymatic shuttles. Into the matrix, NADH can directly transfers electrons to mtCx-I of the ETC.

In contrast, the redox pair NADPH/NADP^+^ is in a more reduced state [112] to provide electrons in particular for reductive biosynthesis. It is in fact known that NADPH acts as an electron donor in anabolic as well as antioxidative reactions, such as the reduction of GSSG to the active antioxidant GSH [113,114]. The replenishment of NADPH, which is necessary to maintain a sustainable NADPH/NADP^+^ ratio, is mainly achieved via NADPH-regenerating enzymes [112]. The activity of these enzymes, which include two enzymes of the oxidative part of the PPP, i.e., G6PDH and 6-phosphogluconate dehydrogenase, the cytosolic NADP^+^-dependent isocitrate dehydrogenase (ICDH) and the cytosolic malic enzyme (ME), is strictly dependent on cell metabolic state.

As far as the cytosolic isoforms of ICDH and ME are concerned, they can produce NADPH when the metabolic state of the cell allow the withdrawals of isocitrate and malate from citric acid cycle, thus contributing to the synthesis of fatty acids and cholesterol by supplying NADPH. Conversely, during acute oxidative stress, the increase in demand for NADPH is guaranteed mainly by G6PDH, a PPP enzyme, whose complete deficiency is incompatible with life, which utilizes the glucose-6-phosphate continuously produced during the first reaction of glycolysis. Noteworthy with regard to mitochondria, nicotinamide nucleotide transhydrogenase, in a reaction driven by the mitochondrial electrochemical proton gradient, can also produce NADPH from NADP^+^ by using the NADH derived from TCA cycle as substrate. Consistently, whenever matrix NADH level or mitochondrial membrane potential decrease, due to mitochondrial malfunctioning, also mitochondrial NADPH regeneration will be impaired, leading to oxidative stress [115]. 

Furthermore, NADPH can be produced by other mitochondrial dehydrogenases, such as mitochondrial isoforms of ICDH and malate dehydrogenase (MDH) [115] and it is also involved in the reaction of the GSH- and thioredoxin-dependent antioxidant enzymes, either cytosolic or mitochondrial, where it participates as an electron donor. 

Concerning GSH, it strongly exceeds GSSG levels [116] and constitutes a strong antioxidant protective tool against ROS, mainly in lung. The GSH/GSSG ratio is controlled by several parameters and its value depends not only on the rates of GSH synthesis, GSH oxidation and GSSG reduction, but also on the availability of GSH to participate in other metabolic pathways (cellular processes) and on the export of either GSH or GSSG, outside the cells. In healthy cells, the level of GSH is kept higher than the oxidized form GSSG by glutathione reductase (GR) which constantly removes the GSSG produced in basal conditions. On the other hand, under oxidative stress conditions, an increase in GSSG level occurs since reduced glutathione is oxidized via chemical or enzymatic reaction. In the last case, glutathione peroxidase (GPx) reduces H_2_O_2_ to H_2_O by utilizing GSH as an electron donor. GSSG is then reduced back to GSH in the reaction catalysed by GR in the *glutathione cycle*. The capacity to recycle GSH makes this cycle crucial to the cellular antioxidant defence mechanism and prevents depletion of thiols. Besides to the glutathione cycle, the GSH/GSSG ratio is also influenced by the effective availability of GSH inside the cell since it that can be exported outside as such in the oxidized form or after conjugation [112].

The picture that emerges highlights that the three cytosolic redox pairs NADH/NAD^+^, NADPH/NADP^+^ and GSH/GSSG could to be more crosslinked than supposed. Indeed, NAD kinase catalyses the production of NADP^+^ from NAD^+^ but NADP^+^ can also be hydrolysed to NAD^+^ [117]. The GSH/GSSG pair is modulated by GR, a NADPH-consuming enzyme, while glucose-6-phopshate is the substrate for both glycolysis and PPP, two metabolic pathways responsible for the production of NADH and NADPH, respectively. Any alterations of one of these redox pairs will produce effect also on the other and, indeed, G6PDH overexpression induces NADPH, NADH and GSH level increase [118] while inhibition of GR reduces GSH but increases both NADH/NAD^+^ and NADPH/NADP^+^ ratios [119].

### 4.3. GSH as Tool to Combat Ox Stress (Infections) 

Since its role in the CF is dominant, more information about GSH concerning its synthesis and use are necessary.

GSH is normally present at 2–10 mM concentrations inside cells. It is synthesized *de novo* exclusively in cytosol—where γ-glutamylcysteine synthase (γ-GCS) and glutathione synthetase (GS) reside—from its constituent amino acids by two successive ATP-dependent enzymatic steps. 

In the first step, cysteine (CYS) and glutamate are linked in a reaction catalysed by the γ-GCS to form γ-glutamylcysteine. This first reaction is the rate-limiting step in the synthesis of GSH and is regulated by CYS availability. About, the antioxidant function of GSH is proper determined by the redox-active thiol (-SH) of CYS that becomes oxidized when GSH reduces target molecules [120]. The completion of GSH synthesis is catalysed by GS, in a reaction in which γ-glutamyl-cysteine is covalently linked to glycine. 

Cytosolic GSH is then distributed among the intracellular organelles including the mitochondria, endoplasmic reticulum (ER), and nucleus—which do not possess the enzymatic machinery to perform *de novo* synthesis of GSH—to control compartment-specific needs and functions [120]. 

Except for the ER, intracellular GSH is mainly found in its reduced form. In particular, although mitochondrial GSH represents about 10% of the total cellular GSH pool, however, based on the volume of the mitochondrial matrix, its concentration is similar to that found in the cytosol: it is estimated to be about 10–14 mM (see [121]).

In addition, γglutamyltransferase (γGT), which is located on the outer surface of the plasma membrane, can degrade extracellular GSH. GCS and γGT constitute part of a system for transport of GSH between organs and for its recycling between the extracellular and intracellular compartments. As a consequence of GSH export by epithelial cells, GSH is found in high concentration in some extracellular fluids, such as the ASL [122]. Normal human ASL contains a high GSH concentration (i.e., 400 μM) that is 140-fold higher than that in the plasma [123]. Extracellular GSH can serve as a scavenger of carbon-centred free radicals produced by lipid peroxidation and hypochlorous acid produced by neutrophils during inflammation. However, it would be wrong to view GSH only or even most importantly in terms of its antioxidant properties when considering its importance in lung defence. Indeed, a second property of reduced glutathione that should not be overlooked is its promotion of mucolysis. Because of its chemistry, GSH, like N-acetylcysteine (NAC), is able to cleave disulphide bonds, which serves to reduce the viscoelasticity of mucus when the GSH system is functioning normally [124].

## 5. Mitochondria in CF: What Is Known?

To date, the involvement of mitochondria in CF has never been investigated in detail.

The paucity of information is mainly due to the fact that after the primordial suspicion that the mutated protein responsible for the disease was of mitochondrial origin, the researchers did not anymore take into account the hypothetical involvement of mitochondria, centering the main research essentially on the protein encoded by the mutated *cftr* gene (see [125]).

Here we review all the studies concerning the involvement of mitochondria in CF, with particular attention to the more recent ones on the altered mitochondrial function. We believe this effort a necessary starting point to obtain a clear and fruitful overall picture that could help to properly address future studies aimed to clarify the molecular mechanisms of mitochondrial dysfunction in CF.

### 5.1. What Was Already Known about Mitochondria in CF?

That mitochondrial defects could somehow related to CF pathogenesis was first hypothesised in 1979 when mtCx-I impairments was reported [125]. In this study, Shapiro and collaborators sustained that in CF cells oxygen consumption increased and the mtCx-I inhibition by rotenone (ROT) was more effective than in normal cells [125]. Furthemore, treatment with ouabain, an inhibitor of the Na^+^-K^+^-exchanging ATPase, was able to reverse the increase in mitochondrial oxygen consumption, thus suggesting that an increase in Na^+^K^+^ATPase activity also occurred to fulfil the energy demands by CF cells [126]. Consistently, about a 50% increase in oxygen consumption was described in epithelial cells derived from nasal polyps in CF patients with respect to control samples. As a consequence, for the increased oxygen consumption by CF cells, the mitochondrial production of both superoxide (O_2_^−•^) and peroxide (H_2_O_2_) could increase too [127].

NADH dehydrogenase also showed differences in enzyme kinetics with decreased Km and increased pH optima in CF cells [128], suggesting that the CF-mutant gene might be responsible for the observed mtCx-I alterations [129]. Moreover, it has been reported that in CF fibroblast also cytochrome-c oxidase showed an altered kinetics with increased Km at temperature >25 °C [130]. Other mitochondrial abnormalities have been described in F508del-CFTR cells such as fragmentation of the mitochondria network and reduction of mitochondrial Ca^2+^ uptake, both events presumably linked to a primary mitochondrial membrane depolarization [131]. Taken together, these findings pointed out to an involvement of mutated CFTR into the impairment of mitochondrial structure and function.

Afterwards, several studies described other mitochondrial changes in CF [131] to such an extent that it was initially thought that the mutated protein responsible for the disease was a mitochondrial protein. However, when CFTR was cloned and identified as a chloride channel [131], the hypotheses of possible mitochondrial involvement in CF was totally put aside to the point of concentrating the whole study on the mutated protein forgetting that the cell lives thanks to the presence of the mitochondria. Therefore, the subsequent works concerned mainly the CFTR as chloride channel. Only few studies continued to explore mitochondrial involvement in CF. In particular, it was proven that the 2D electrophoretic patterns of mitochondrial proteins was proved to be different in CF patients with respect to controls [132], as well as intracellular pH increased in CF subjects during workload [133].

Besides being a chloride channel, and as such clearly endowed with a transport activity, the CFTR can also indirectly affect gene expression. It has been reported that some CFTR-dependent genes are involved in specific cell pathways—either metabolic or inflammation-related [124]—and two genes in particular, MT-ND4 and CISD1 encoding for mitochondrial proteins, are downregulated in CF cells [131].

In particular, MT-ND4 gene encodes for one of seven subunits of the mtCx-I, the ND4 subunit. It is crucial to the proper assembly and activity of mtCx-I [131,134,135]. As a consequence, the MT-ND4 downregulation detected in CF cells could be responsible for the low efficiency in NADH oxidation. Indeed, as also discussed below (see Section 5.2.1), the activity of mtCx-I decreased in CF cells (see [131,136]) and it should be considered that, as suggested by Cleeter et al. [137], a deficient mtCx-I may increase the level of ROS, which in turn further affects mtCx-I activity. Conversely, inhibition of the OXPHOS system, described by Esposito et al. [138] in the Ant1(tm2Mgr) (-/-) mouse model which is depleted of the heart/muscle isoform of ANT, induced ROS production as well as the expression of manganese superoxide dismutase (Mn-SOD or SOD2) as a compensatory mechanism [131]. These conditions apparently in antithesis indicate that both the origin and the consequences of high ROS levels are not fully understood. 

Whenever the antioxidant system fails to balance the increasing ROS, a damage to mtDNA could easily occur which further impairs the OXPHOS system thus inducing a vicious cycle of additional ROS generation [131]. Such findings have been confirmed in human RPE cells by Lian and Godley [139]. A mitochondrial impairment, due to increasing oxidative stress, has also been described in CFTR-knockout mice where both an oxidative damage to mtDNA and a reduced aconitase activity have been observed [121].

There are several factors that make CF cells more prone to injury by oxidative stress, and an altered GSH/GSSG ratio is the first among them. As already reported (see Section 4.3), GSH is a key antioxidant compound whose availability inside cell is fundamental to sustain a good redox state and the health of cells. In CF, the low CFTR activity has been correlated to a defective GSH transport [140,141] (see above, Section 2.1.3) resulting in an altered extracellular ratio between reduced and oxidized glutathione [131]. 

Altered GSH level in CF has already been reported in the initial studies done in the 1970s [121]. Consistently, transfection of normal CFTR has been reported to result in increased GSH [114]. Concerning this, Kelly-Aubert et al. [142] reported that the treatment with a membrane permeable analogue of GSH, i.e., GSH monoethylester (GSH-EE), reverted the reduced mtCx-I activity of CF cells, as well as CFTR knockout mice, to healthy values. Likewise, also the ΔΨ was restored by GSH-EE. Taking into account that GSH-EE was found to be able to increase the levels of mitochondrial GSH (mGSH) in different experimental models (see [131]), it clearly emerges that the GSH depletion is a predisposing factor to mitochondrial dysfunction in CF cells. Either in liver or in neurodegenerative disorders, such as Parkinson’s and Alzheimer’s disease, mGSH depletion has been correlated with alterations of the respiratory chain [142] in particular of mtCx-I the more likely among the respiratory complexes to be inactivated by ROS and/or by GSH/GSSG variations [143].

Any attempt for defining the cause–effect relationship between mtCx-I inhibition and GSH depletion, in order to define which mechanism comes first, is still waiting for an answer. What is known is that each mechanism causes an increase of level of ROS, which in turn modify the GSH/GSSG ratio by consuming GSH and lead to mtCx-I inhibition due to oxidative modifications. mtCx-I inhibition and mtGSH depletion are interconnected in a round loop fuelled by ROS elevation.

Notwithstanding GSH treatment, either by inhalation or oral administration of GSH or NAC, has been administered to CF adults and children enrolled in several clinical trials [144], none of them proved to be really effective in reduce sputum elastase activity and IL-8 levels while a short-term administration only slightly improved lung function. Moreover, GSH-EE was able to re-establish suitable levels of mGSH and to correct the cellular damage [145], but it was found to be toxic at high doses probably as a result of the ethanol production occurring when GSH is released [146]. At present, this issue limits its use in vivo.

Next, taking into account what has been reported so far, we will review the recent findings on mitochondrial alterations found in CF cells and their possible pathophysiological consequences.

### 5.2. The Latest Findings on Mitochondria in CF

From the above, one thing is certain: mitochondria have an enormous potential to influence health. This leads us to firmly believe that optimizing the metabolism of mitochondria in those diseases, such as CF, in which mitochondrial function is compromised, can be the focus of effective treatment therapeutic. 

The latest studies to which we will refer in this section started about five years ago.

We approached the study of mitochondria in CF taking into account two assumptions: (i) oxidative stress plays a pivotal role in the pathogenesis of CF [131] and (ii) mitochondria play a major role in cellular redox homeostasis [147,148,149]. 

Aim of our study has been to find the intertwined relation between F508del-CFTR and mitochondrial bioenergetics, with respect to both oxidative stress and redox imbalance in-order-to describe some features of the complex CF phenotype and detect potential new targets for therapy.

#### 5.2.1. Characterization of Mitochondrial Function in Cells with Impaired CFTR Function 

First, the principal goal has been to investigate mitochondrial function, in particular as it regards the steps of OXPHOS and ROS production, in airway cells. In this regard, experiments concerning this research were made using two human bronchial epithelial cell lines: CFBE41o- cells expressing F508del CFTR and respective control, i.e., CFBE41o-cells stably expressing wildtype CFTR. For convenience these cells will be referred to as ‘CF cells’ and ‘control cells’ in the text.

We observed that some steps of OXPHOS, such as ADP/ATP exchange via ANT, oxygen consumption, ΔΨ generation and both mtCx-I and COX, activities are impaired in airway cells homozygous for the F508 deletion, while both ROS production and mitochondrial membrane lipid peroxidation increased [136] (Figure 2). 

In particular, we found a loss of mtCx-I activity with consequent ROS increase. Further, we proved that ROS-mediated damage of the membrane microenvironment was likely responsible for inhibition of COX, whose activity is strongly dependent on the membrane lipid environment (see Figure 2). Importantly, treatment of CF cells with the small molecules VX-809 and 4,6,4’-trimethylangelicin (TMA), which act as ‘correctors’ for F508del CFTR by increasing the amount of functional CFTR at the cell surface and rescuing the F508del CFTR-dependent chloride secretion [61,62] (see above, Section 2.2.2), significantly improved all the mitochondrial parameters towards values found in the airway cells expressing wildtype CFTR, strongly suggesting that the restorative action provided by the correctors on mitochondrial functions in CF cells is linked to the rescue of chloride channel activity. Unfortunately, we could not currently provide any molecular mechanism underlying how CFTR dysfunction affects parameters of mitochondrial function, nor how corrector-induced increased CFTR cell surface expression is able to repair these mitochondrial dysfunctions. 

At the same time, we obtained the same results by using as a model study primary cells, which provide a microenvironment closer to in vivo situations.

These results were valuable because they represented the starting point to address the next research. Indeed, since (i) the mitochondrial dysfunction and ROS generation are intricately related to changes in the glutathione redox system [147]; (ii) a drop of GSH levels is observed in CF cells [81,150], we studied more precisely GSH and GSH-dependent enzymes in order to trace back the link between mitochondrial dysfunction, low GSH levels and defective F508del-CFTR.

In particular, the research was devoted to:

- Detecting the enzyme/s contributing to the upregulation of intracellular ROS production, besides mitochondria [131,136];
- Studying how the balance between the production and neutralization of ROS is maintained in the presence of antioxidant enzymes, measuring the activity of superoxide dismutase (SOD) and catalase;
- Measuring both the GSH-dependent enzyme, i.e., GPx and GR activities, and the GSH levels, either inside or outside the cell;
- Analysing the redox states of the NAD and NADP pyridine nucleotide pools, which play critical roles in defining the activity of energy producing pathways and in both driving oxidative stress and maintaining antioxidant defences, respectively;
- Identify the involvement of CFTR—if any—as part of the GSH cycle.

It is noteworthy that the objective was not to study changes in enzymatic activities and/or metabolite levels, but to understand the interaction dynamics existing between enzymes and levels of metabolites/cofactors.

The findings, i.e., the increased production of ROS is crucial to the progression of CF [131] and, consistently, the high levels of lipid and protein oxidation products found in bronchoalveolar lavage fluid of CF patients [142], prompted us to investigate further the origin of ROS in CF, besides those coming out by mitochondria activity.

#### 5.2.2. Defective CFTR and NOX/GR Activity Imbalance Contribute to ROS Overproduction

Together with mitochondria [136], NOX was the prominent source of ROS, as revealed by the ability of its inhibitor Diphenyliodonium (DPI) to drastically lower O_2_^−•^ level in cells. Moreover, that NOX preferentially uses NADPH over NADH as an electron donor turned out to be extremely interesting—if you think that NADPH mainly plays an important role in fighting oxidative stress (see below). This conclusion was strengthened by NADPH oxidase protein overexpression [116]. Consistently, it is largely known that increased oxidative stress and enhanced ROS production [151] may largely originate from enhanced and/or inappropriate NOX activation in chronic diseases of the respiratory tract, such as COPD, asthma, CF, or in various forms of lung cancer.

However, considering that excessive levels of extracellular and intracellular ROS may result from increased ROS production but also from defective cellular antioxidant (AOX) system, the authors—in the same study—showed a 50% decrease of GR activity, probably due to post-translational enzyme modification since GR protein level remained unchanged. 

Then, we are dealing with a perturbation of the equilibrium between two enzymes working in opposition, i.e., NOX, requiring NADPH to produce O_2_^−•^, and GR, using NADPH to restore GSH levels, with NADPH being probably channelled preferentially towards NOX rather than GR reaction. In order to confirm that really GR and NOX are competing for cytosolic NADPH, it was observed that GR reaction rate increased in CF cells incubated with NOX inhibitor DPI (Figure 2).

Interesting to note that though an increase of SOD activity—but not of catalase and GPx—was found in CF cells, a slight increase of ROS level was detected in the presence of SOD inhibitor, suggesting a negligible action of this enzyme in protecting CF cells against pro-oxidant insults.

Bounteous of information, useful to our research aim, was the study on the modulation of the ratios of the redox-active cofactors NADH/NAD^+^, NADPH/NADP^+^ and GSH/GSSG which hit cell metabolism, an unexplored realm in the search for CF.

Under normal conditions—as reported above (see Section 3.2)—the NADH/NAD+ pair is predominately in the oxidised state (see above); in contrast, the redox pairs NADPH/NADP^+^ and GSH/GSSG are biased towards the reduced state to supply electrons for reductive biosynthesis and antioxidative processes, respectively. 

An overturned situation was found in CF: the cytosolic redox state of the NADH/NAD+ pair was inclined to the reduced state, whereas the NADPH/NADP^+^ pair to the oxidized one’s. These results confirmed the reduced mtCx-I activity to oxidise NADH due to low OXPHOS [129] and, in addition, they suggested that the reduced quantity of NADPH, the main cellular reducing equivalent required by many antioxidant defence systems [152,153], could be responsible, totally or in part, for the low intracellular GSH (inGSH) level (see below) and then have a profound effect on ROS levels in CF cells. In this regard, to avoid getting in the experimental details, we invite the reader to read how the experiments were made as well as the strategic procedure adopted in order to understand the mechanism by which CFTR modulates extracellular GSH (exGSH) level in CF airway cells. 

Regarding GSH levels, both extra- and intracellular, this issue merits some considerations. Indeed, since exGSH level is low and it depends on its impaired transport across plasma membrane due to deficient CFTR function, this suggests that contrariwise inGSH increased. But, surprisingly— and contrary to expectations—we found a significant decrease in inGSH content in CF cells, in accordance with [154], which was largely prevented by VX-809-treatment (about 100%). 

Then, investigating on the inGSH level which depends upon the equilibrium between its consumption and biosynthesis, the latter process being limited by CYS availability [150,155], we guessed that the CYS could have a role in this dynamics. To confirm this, an increase of inGSH (about 50%) was found when CF cells were preincubated with CYS, suggesting that when CYS is available outside the cells, it is used for intracellular GSH synthesis (see [156]) (see Figure 2). The hypothesis that we advanced thanks to the obtained experimental observations was that a low exGSH amount, consequent to the CFTR deficit, can contribute to the decrease of inGSH level due to a reduced CYS regeneration by γGT (see Figure 2). In support to this hypothesis, we found that: (*i*) treating CF cells with Acivicin (ACI), specific γGT inhibitor [157], the inGSH level further decreased, even below that obtained in untreated CF cells, but it recovered up when cells were treated with ACI plus CYS; (*ii*) treating normal cells with CFTR(inh)-172, a specific inhibitor of CFTR channel [158], the inGSH level decreased of about 40% with respect to the inGSH level of untreated cells and it was almost completely restored when CYS was added together with CFTR(inh)-172.

What has been said so far leads to firmly setting salient points: (*i*) in CF cells some steps of OXPHOS are impaired, with both mitochondrial ROS production and membrane lipid peroxidation increase; (*ii*) ROS overproduction is also due to increased NOX activity; (*iii*) the overt oxidative stress condition elicits the loss of cell redox balance—a condition which sees the involvement of GSH in the front row—with deleterious consequences for metabolic regulation.

This should be kept in mind, especially in light of what will be described in the next paragraphs. Starting from the observation that the high ASL GLU concentration in human patients with CF [87] is responsible for the burst of the lung infection by pathogens [159], together with the highly expression of CFTR in the airway epithelium, it is reasonable to think that a CFTR defect leads to changes in the ASL lining the lungs, causing poor clearance of bacteria which ultimately exacerbates inflammation (see [39,160]). 

The current model for airway GLU homeostasis assumes that the concentration of GLU in the ASL is the net effect of paracellular diffusion (and, to a lesser extent, the transcellular flux of GLU) from the blood and interstitial fluid across respiratory epithelium into the ASL and removal of GLU from ASL by GLU transporters (GLUTs) and cellular metabolic enzyme/s [161]. In this context, a metabolomic approach had revealed that the levels of glucose and various glycolytic intermediates were significantly reduced in CF cells [148]. Furthermore, increased activity of four glycolytic enzymes in cultured fibroblasts from CF patients was found [162], whereas in 1981, researchers found a G6PDH deficiency in CF [131]. 

Our new research path aimed to investigate some of the thousands of metabolic reactions (see Section 5.2.3)—those related to glucose metabolism and the production of NADPH—with the ultimate aim to restrict GLU availability in the ASL, action of extreme importance in order to control lung infection by pathogens. 

With the term ‘cellular metabolism’ refers to the complex set of chemical reactions that permit cells, organs, and entire organisms to function and thrive. Although cellular metabolism is often discussed in the context of individual pathways, survival of an organism is ultimately dependent on the integration of all metabolic pathways. In fact, other than glycolysis, no major metabolic pathway functions entirely on its own; for example, the PPP most commonly relies on G6P from glycolysis in order to proceed, and lipid synthesis cannot move forward without input of both NADPH and ATP from at least two other metabolic pathways. While numerous other examples could be highlighted, the major point is that biochemical events in one metabolic pathway cannot be easily understood if discussed only in isolation.

According to a logical assumption, we all were agreed that the extracellular GLU-lowering action exerted by cell membrane transporter/s and cytosolic enzymes was necessary, but perhaps not sufficient. Biochemical approaches have allowed to respond to questions about how G6P is partitioned between glycolytic and PPP and whether the PPP, appropriately controlled, plays a crucial role against oxidative stress in CF cells. Strategic manipulations of both GLU-utilizing pathway enzymes, i.e., Glycolysis and PPP, and mitochondrial function proved useful for understanding how the cells could fight the high load of ASL GLU and ROS in CF.

#### 5.2.3. Modulation of Glucose-Related Metabolic Pathways Helps both Reduce Glucose Level in ASL and Fight Oxidative Stress

Lung epithelial cells are able to oxidize GLU to produce energy (see Section 3.4). The availability of intracellular GLU is under the control of GLUTs that not only control its movement across the lung epithelium, but are also involved in regulating GLU level in ASL. Once inside the cell, GLU is immediately metabolized by cytosolic enzymes (for detail see above, Section 3.1 and Section 3.4). 

Considering that the metabolic pathways of glycolysis, Krebs cycle and respiratory chain are tightly interconnected, it becomes easy to realize that any alteration in mitochondrial respiration (see [125,136]) or in the processes regulating GLU uptake and utilization, inevitably involve both mitochondrial and cytosolic metabolic pathways, mutually (see Figure 2). 

As far as the GLU metabolism in airways cells is concerned, the first step is the uptake across the cell membrane. In this regard, Garnett et al. [92] have demonstrated that in human H441 airway cells the levels of GLU2 and GLUT10 can be modulated by pro-inflammatory stimuli. Consistently, it has recently been demonstrated that not only GLUT activity increases but also the protein level of GLUT1, the most ubiquitously expressed isoform of GLU transporter in humans (see [161]), is upregulated. However, the overall upregulation of GLU transport seems not to be sufficient to prevent the rise of GLU concentration in ASL. This does not exclude a priori the possibility to intervene on a mechanism that could dynamically regulate the ASL GLU level as it increases during inflammation. 

In addition to GLUT, also the activity of the two most important glycolytic enzymes, HK and PFK, increased in CF cells, as well as their protein levels although to a lesser extent. Similarly, in fibroblasts from CF patients, the increase in the activity of four glycolytic enzymes was detected [162].

Different was the situation for G6PDH, a key enzyme in regulating the GSH availability and ensuring protection against cellular ROS in healthy cell. Enzymatic activity and protein level of G6PDH decreased in CF cells as compared to control cells. These findings strongly support the close relationship between NADPH and GSH level decrease (see above) and G6PDH decrease in CF cells where ROS level increased [116].

Recently, a deep investigation has been carried out as to whether a relationship exists between the redox state of the cell and the GLU metabolism both inside the cell and in ALS, taking advantage of a set of compounds able to modulate G6P utilization and glycolytic ATP production. 

It was found that in the presence of 6-aminonicotinamide (6AN), G6PDH inhibitor, the level of G6P was almost doubled while ROS levels were reduced by a half, thus suggesting that (*i*) in CF cells G6P is preferentially metabolized through PPP and (*ii*) PPP-derived NADPH is likely to be the driving force to generate NOX-derived ROS, being the latter an enzyme whose activity overcomes that of GR [116] (see above, Section 5.2.2).

Unexpectedly, G6P and ASL GLU levels were unchanged in CF cells in the presence of CITR, an anti-glycolytic agent that inhibits PFK, thereby largely inhibiting phosphorylation at the substrate level and slowing glycolysis. This singular effect suggests that when PFK is inhibited by CITR, glycolytic flux is ‘gated’, as confirmed also by the collapse in L-LAC level. In this condition, G6P reaches a sort of ‘steady-state’ level being able to inhibit HK from one side, so that no further production of G6P occurs, and to be metabolized by G6PDH along PPP on the other side. Thus, in the presence of CITR, the cell is forced to produce more NADPH, also thanks to a more active G6PDH, in a sort of compensatory mechanism for the inhibited activity of glycolysis and the reduced mitochondrial ETC (see above, Section 5.2.1).

Surprisingly, although the NADPH level increased, the ROS level actually decreased in the presence of CITR in CF cells, suggesting that the increase in NADPH level in the presence of CITR was not able to accelerate NOX activity, thus confirming the hypothesis that the point at which the glycolytic flow is blocked—by CITR—is crucial for the regulation of cell redox status in CF. These results agree with the observations that a high NADPH level is required in CF in response to infection [152] as well as in the temptative to counteract the ongoing oxidative stress [125,131,136].

When the activity of mitochondrial respiratory chain is inhibited at level of Complex I and IV, i.e., in the presence of ROT+OLIGO, a low level of G6P but an increase in L-LAC are detected and these results can be easily explained as an extreme tentative of the cell to upregulate the glycolytic enzymes metabolizing G6P to prevent its accumulation, consistent with Glycolytic index (GI) increase. 

Interestingly, GI values, per se higher in CF as compared to control cells (3.1 versus 0.8), further increased when CF cells were treated with ROT+OLIGO confirming that when residual mitochondrial activity is inhibited, cell metabolism strictly depends on the anaerobic glycolytic pathway.

We are facing a dizzying situation: the reduction of mitochondrial respiration seems to be advantageous for the reduction of GLU of ASL and also for the reduction of the level of ROS in CF cells. To disentangle ourselves in this complex matter, with the aim to interpret the metabolic environment of ASL in CF, two models of disease (Alzheimer’s disease and cancer) in which cooperation was observed between mitochondria and glycolytic enzymes have intervened [163].

Due to the close co-operation between cytosolic metabolism, i.e., glycolysis, and mitochondria, it is understandable that when glycolysis is inhibited at the level of PFK, the supply of pyruvate to mitochondria will be reduced with consequent reduction in mitochondrial activity. Indeed, in the presence of CITR, i.e., when mitochondria functions are repressed, the general conditions of CF cells seem to be improved both inside as well outside.

Accordingly, in CF cells treated with CITR, and even more with ROT+OLIGO, the ROS level decreased thus suggesting that ROS-dependent mitochondrial metabolism is central to disease as well as a crucial element of the CF phenotype. On the other hand, the findings that both upregulation of glycolytic enzymes and downregulation of G6PDH, occurring in CF cells, did not reduce ASL GLU clearly suggest that mitochondrial activity has a prominent role in CF cells and, thus, only a low efficiency of mitochondria may restrain the progressive impairment of CF cells.

In conclusion, when the mitochondria are quiescent, i.e., when mitochondrial activity is below a certain threshold value—mitochondria dictate the conditions in which the cell is, having a beneficial effect detectable in the lowering of both ROS and ASL GLU levels, responsible of infection by pathogens in CF.

## 6. Conclusion Remarks

As it is clear from the discussion above, mitochondrial functions extend beyond the boundaries of the cell and influence an organism’s physiology by regulating communication between cells and tissues. It is therefore not surprising that mitochondrial dysfunction has emerged as a key factor in a myriad of diseases.

Then, the research field aimed at “targeting mitochondria” is active and expanding.

It goes without saying that one of the important objectives of managing patients with mitochondrial disorders is to prevent drugs from being toxic to mitochondrial functions. In fact, drugs can affect many of the different functions within the mitochondria. Drug therapy-induced ETC dysfunction may result from the direct inhibition of one or more of the enzyme complexes or uncoupling of OXPHOS. As the enzyme complexes are susceptible to free radical-induced oxidative damage, drugs that cause oxidative stress may also result in ETC toxicity. 

Significant progress has been made over the last several decades in understanding of energy metabolism in the lung. Recent technological advances have enabled researchers to go beyond studying just whole organ metabolism and begin dissecting the metabolic events driving common and unique behaviours in individual cell populations in the lung. Although the pulmonary community has made significant progress, understanding of pulmonary metabolism still lags behind that of many other fields. 

In order to deal with these problems, it will need to invest more heavily in the field, including taking advantage of recent forefront technologies with the wish to yield new biological insights and also to identify previously unrecognized biological markers that can aid in the diagnosis, screening, and/or monitoring of respiratory diseases. Understanding the molecular mechanisms regulating the mitochondrial function of lung cells will help to better define phenotypes and clinical manifestations associated with respiratory diseases and to identify potential diagnostic and therapeutic targets.

Regarding CF, what has been described currently is a pure basic research study, but investigating a poorly explored and undoubtedly interesting topic, i.e., the optimizing of the mitochondrial metabolism knowledge, could prove to be valuable in the future, reaching the focus of an effective therapeutic treatment and assisting the CF patient.

As such, we maintain that it would be interesting if mitochondrial functions were studied in other cells, such as macrophages and neutrophils, considering the importance these cells have in the progression of the disease, as briefly described above (Section 2.1.4).

## Abbreviations

6AN	6-aminonicotinamide
ABC	ATP Binding Cassette
ACI	Acivicin
ANT	Adenine nucleotide translocase
AOX	Antioxidant system
ASL	Airway surface liquid
ATP	Adenosine 5′-triphosphate
CF	Cystic Fibrosis
CFTR	Cystic Fibrosis Transmembrane Conductance Regulator
CITR	Citrate
COPII	Protein Complex II
COX	Mitochondrial Complex IV
CYS	Cysteine
DPI	Diphenyliodonium
ΔΨ	Mitochondrial membrane potential
ENaC	Epithelial sodium channel
ER	Endoplasmic reticulum
ETC	Electron transport chain
exGSH	Extracellular GSH
GI	Glycolytic index
GLU	Glucose
GLUT	Glucose transporter
G6P	Glucose-6-phpsphate
G6PDH	Glucose-6-phosphate dehydrogenase
GPx	Glutathione peroxidase
GR	Glutathione reductase
GSH	Reduced glutathione
GSSG	Glutathione disulphide
CYS	Cysteine
γ-GT	γ-glutamyltransferase
γ-GCS	γ-glutamylcysteine synthase
HK	Hexokinase
H_2_O_2_	Hydrogen peroxide
ICDH	Isocitrate dehydrogenase
IMS	Intermembrane space
inGSH	Intracellular GSH
L-LAC	L-lactate
MDH	Malate dehydrogenase
ME	Malic enzyme
mtCx-I	Mitochondrial Complex I
mGSH	mitochondrial GSH
Mirs	microRNAs
NAC	N-acetylcysteine
NOX	NAD(P)H oxidases
O_2_	Molecular oxygen
O_2_^−•^	Superoxide anion radical
OLIGO	Oligomycin
OXPHOS	Oxidative phosphorylation
PFK	Phosphofructokinase
PKA	Protein kinase A
PPP	Pentose phosphate pathway
ROS	Reactive oxygen species
ROT	Rotenone
SOD	Superoxide dismutase
TCA	Tricarboxylic acid cycle
TMA	4,6,4′-trimethylangelicin
